# A new method for detecting the outer corneal contour in images from an ultra-fast Scheimpflug camera

**DOI:** 10.1186/s12938-019-0735-1

**Published:** 2019-12-03

**Authors:** Magdalena Jędzierowska, Robert Koprowski, Sławomir Wilczyński, Katarzyna Krysik

**Affiliations:** 1 Institute of Biomedical Engineering, Faculty of Science and Technology, University of Silesia in Katowice, ul. Będzińska 39, 41-200 Sosnowiec, Poland; 2 Department of Basic Biomedical Science, School of Pharmacy with the Division of Laboratory Medicine in Sosnowiec, Medical University of Silesia in Katowice, Kasztanowa Street 3, 41-200 Sosnowiec, Poland; 3 Department of Ophthalmology with Paediatric Unit, St. Barbara Hospital, Trauma Centre, Plac Medykow 1, 41-200 Sosnowiec, Poland

**Keywords:** Edge detection, Corneal, Corneal contour, Roberts, Sobel, Canny, Image processing, Corvis ST, Scheimpflug camera, Tonometry

## Abstract

**Background:**

The Corvis^®^ ST tonometer is an innovative device which, by combining a classic non-contact tonometer with an ultra-fast Scheimpflug camera, provides a number of parameters allowing for the assessment of corneal biomechanics. The acquired biomechanical parameters improve medical diagnosis of selected eye diseases. One of the key elements in biomechanical measurements is the correct corneal contour detection, which is the basis for further calculations. The presented study deals with the problem of outer corneal edge detection based on a series of images from the afore-mentioned device. Corneal contour detection is the first and extremely important stage in the acquisition and analysis of corneal dynamic parameters.

**Result:**

A total of 15,400 images from the Corvis^®^ ST tonometer acquired from 110 patients undergoing routine ophthalmologic examinations were analysed. A method of outer corneal edge detection on the basis of a series of images from the Corvis^®^ ST was proposed. The method was compared with known and commonly used edge detectors: Sobel, Roberts, and Canny operators, as well as others, known from the literature. The analysis was carried out in MATLAB^®^ version 9.0.0.341360 (R2016a) with the Image Processing Toolbox (version 9.4) and the Neural Network Toolbox (version 9.0). The method presented in this paper provided the smallest values of the mean error (0.16%), stability (standard deviation 0.19%) and resistance to noise, characteristic for Corvis^®^ ST tonometry tests, compared to the methods known from the literature. The errors were 5.78 ± 9.19%, 3.43 ± 6.21%, and 1.26 ± 3.11% for the Roberts, Sobel, and Canny methods, respectively.

**Conclusions:**

The proposed new method for detecting the outer corneal contour increases the accuracy of intraocular pressure measurements. It can be used to analyse dynamic parameters of the cornea.

## Background

Tonometry is a technique for measuring intraocular pressure (IOP), which is one of the basic ophthalmologic examinations. Elevated intraocular pressure is one of the main factors that may indicate open and closed angle glaucoma [1]. Increased intraocular pressure can also result from other congenital and acquired eye diseases, ophthalmic surgery and systemic diseases [2]. Goldmann applanation tonometry is the gold standard in measuring intraocular pressure. However, despite widespread availability, this method has some limitations. Measurements are only made at a selected point (on a specific surface) and under local anaesthesia. Moreover, the method is contact oriented and requires aseptic conditions. Currently, it is known that this measurement is influenced by, among others, central corneal thickness (CCT) [3–6], corneal curvature [7], age [8, 9] and biomechanical parameters of the cornea [10–12]. Therefore, new devices are still appearing on the market, which, in addition to IOP measurement, provide a number of additional parameters aimed at presenting the biomechanics of the eye and dynamic corneal deformation that occurs during the measurement [13, 14]. The first device to examine corneal biomechanics was the non-contact tonometer ORA (Reichert Technologies, NY, USA). This device is distinguished by two parameters: corneal hysteresis (CH) and cornea resistance factor (CRF), the use of which has already been widely described in the analysis and classification as well as treatment of eye diseases, among others keratoconus and glaucoma [15–21]. The usefulness of the above parameters has also been described in patients who underwent ophthalmic procedures [12, 17, 22–24]. These parameters allow for the analysis of dynamic corneal deformation during air-puff tonometry tests. Unfortunately, they only provide a point score (at the central point of the cornea) of this dynamic process. Due to the limitations of the ORA tonometer, a new device, presenting a number of innovative parameters allowing for the assessment of corneal biomechanics, appeared shortly after. The device is the Corvis^®^ ST (OCULUS Optikgeräte GmbH, Wetzlar, Germany), which is based on the technology using an ultra-fast Scheimpflug camera combined with a classic non-contact tonometer. The Scheimpflug camera, also available in other devices such as Pentacam (OCULUS), enables accurate corneal imaging, e.g. measuring its thickness or detecting and evaluating its diseases, including corneal opacity [25, 26]. In the Corvis^®^ ST, the air stream directed at the eye is illuminated through a 9-mm gap, and the camera records the movement of the cornea at 4330 frames per second. At the beginning of the measurement, the camera records the image of the cornea in its natural, convex shape. Then, under the influence of an air puff, the cornea changes its shape from convex to concave, passing successively through the first applanation phase (flattening), the highest concavity (HC), and returning to its natural shape, through the second applanation phase [27, 28]. By registering the full process of the corneal movement, it is possible to obtain a number of parameters to assess the dynamics of this process. The ability to observe the course of corneal deformation allows for a much more accurate analysis of corneal biomechanics than in the case of the ORA tonometer. The parameters available in the commercial Corvis^®^ ST tonometer software include: corneal deformation amplitude (DA), central corneal thickness (CCT), lengths of the first and second corneal applanation. These parameters have been frequently examined in numerous studies [29–31]. In recent years, researchers have proposed many new, original parameters describing dynamic corneal deformation based on the analysis of 2D images of the deformed cornea acquired from the Corvis^®^ ST [32–38]. Moreover, additional parameters are available in the latest Corvis^®^ ST tonometer software, which in the literature are commonly referred to as dynamic corneal response (DCR) parameters. They were identified as a result of the analysis of specific stages of dynamic corneal deformation. On their basis, a special indicator, the so-called corneal biomechanical index (CBI), was developed, which intuitively indicates the probability of corneal ectasia in the examined patient.

Analysis of medical images, which include images from the Corvis^®^ ST, is often based on the use of modern algorithms and transformations. Today, various methods dedicated to image analysis are used in this field. Unfortunately, when confronted with real medical images, they turn out to be insufficient in most cases and eventually fail. Therefore, in each case, the algorithm must be individually adapted to the data. This is due to the individual character of each case, i.e. high individual variability of analysed patients, as well as artefacts created in the registration process, characteristic of a given imaging method. Such problems also appear in the analysis of images from the Corvis^®^ ST.

**Fig. 1:**
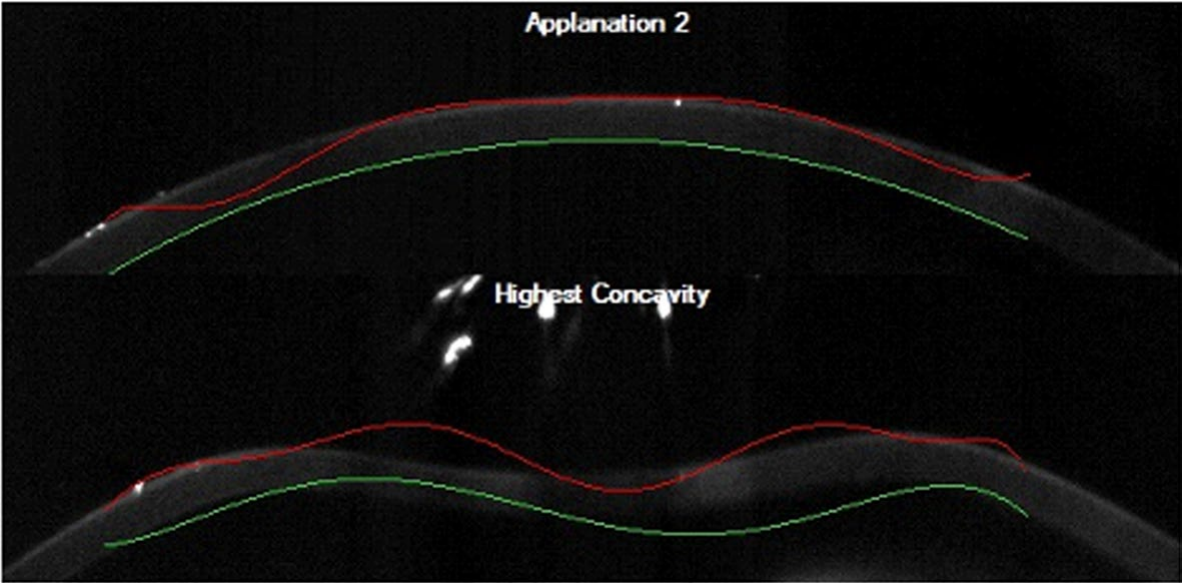
Examples of images from the Corvis^**®**^ ST tonometer showing erroneously detected outer (red line) and inner (green line) corneal edges using the software provided with the device

The impact of individual characteristics and the difficulty in the analysis of images from the ultra-fast Scheimpflug camera can often be seen in problematic images that commercial software provided with the device cannot handle (examples of images are shown in Fig. 1).

The main problem is the erroneously detected outer corneal edge, the determination of which is essential to acquire the characteristic parameters of corneal deformation. Special attention should be paid to the correctness of corneal contour detection, because the detection of its outer edge is the first and necessary step in determining parameters such as:

- Central corneal thickness (CCT). Evaluation of this parameter is particularly important when diagnosing corneal diseases, e.g. keratoconus. Patients with this disorder are characterised by smaller CCT [39].
- Parameters describing corneal vibrations, applicable, among others, in the classification of corneal diseases [40].
- Whole eye movement, which allows for the separation of the entire eyeball displacement from the dislocation of the cornea itself. On this basis, the parameters are divided into those that take into account the displacement of the eyeball, which are described in the literature as deformation parameters, and those that contain only the “raw” corneal displacement, which are described as deflection parameters.
- Other parameters described in the literature, for example, deflection amplitude ratio, highest concavity delta arc length and integrated inverse concave radius [38, 41].

It should be emphasised that the accuracy of the obtained parameters depends on the method of outer corneal contour detection. These results are already used in practice by ophthalmologists in disease diagnostics. In particular, they are used in the early detection of keratoconus [36] and in the assessment of refractive surgery [42]. The mentioned keratoconus is a rare degeneration that makes the corneal centre thinner. Under the influence of intraocular pressure, the cornea becomes cone shaped. The development of this disease causes even more significant bulging of the cornea, it gets thinner and its shape becomes more irregular. Such degenerations are a challenge for scientists, since the corneas of patients suffering from, for example, keratoconus are unique; therefore, the algorithms working properly for healthy patients may fail in those with diseased corneas.

A common problem is the use of polynomial approximation in determining the corneal edge [13, 43, 44]. The use of approximations in a process as dynamic as corneal deformation can result in false edges by marking an area that is not the cornea itself. Therefore, classic edge detection methods often prove to be unsuitable for problems that use real data with high variability.

**Fig. 2:**
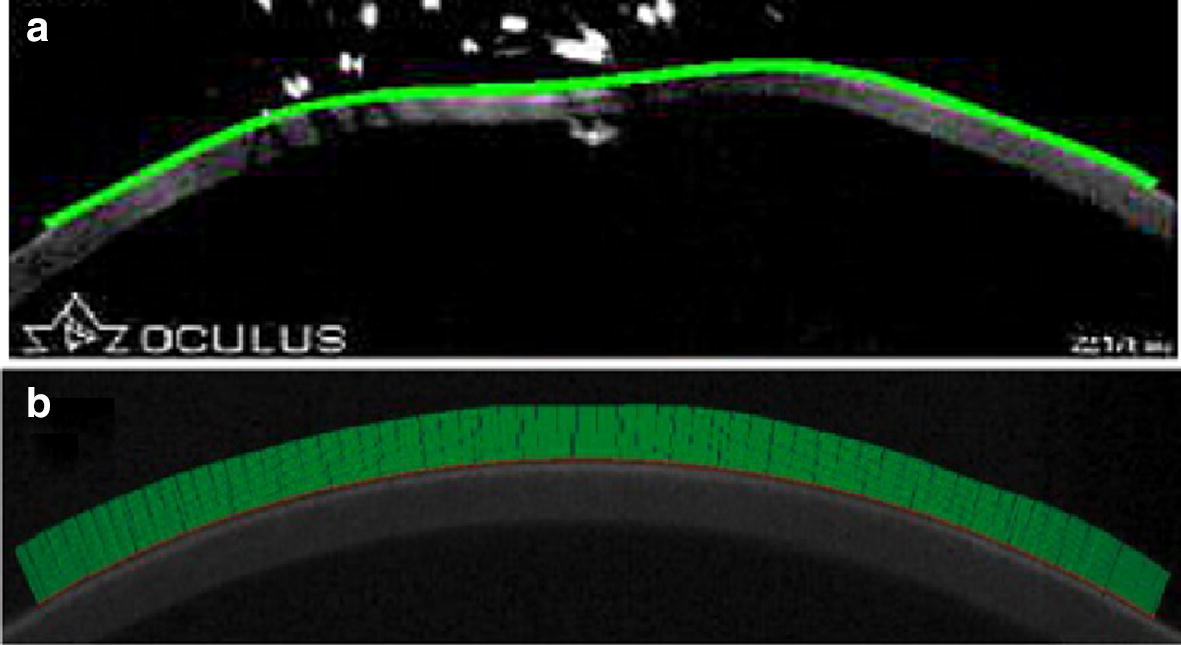
Examples of problems with corneal edge detection described by other authors [13, 43]. **a** An image showing the result of outer corneal edge detection (green line) in the image from the Corvis^®^ ST tonometer using the method presented in [13]. **b** A corneal image before deformation, from the Corvis^®^ ST tonometer, along with the detected outer corneal curvature (upper edge of the green area) based on the algorithm presented in paper [43]

The above problems also appear in the studies of other authors. In the paper by Ji et al. [13], the method used is resistant to small image noise (Fig. 2), but limited by the lack of contour detection at the corneal edges. This solution takes into account the adjustment of the 5th degree polynomial to the corneal edges, which in turn significantly simplifies the described problem and does not allow for accurate consideration of individual corneal variability, especially in the case of patients with, for example, keratoconus. It is also worth paying attention to the study by Kasprzak et al. [43], where the authors use repetitive Gaussian smoothing of the detected, raw outer corneal edge. This approach introduces limitations into the analysis of corneal deformation, especially at the time of the so-called oscillatory phase of corneal deformation.

The problem with corneal contour detection is related not only to Scheimpflug camera images, but also to images from other devices, e.g. OCT (optical coherence tomography). The obtained contour allows ophthalmologists to measure corneal thickness or its curvature radius. These measurements are useful in the diagnosis of patients and refractive surgery. Segmentation and isolation of the cornea profile from many cross sections also allow for the creation of corneal models useful from the point of view of numerical simulations [45].

Given the above, this paper presents the original method of outer corneal edge detection on the basis of a series of images from the Corvis^®^ ST tonometer. In its assumption, the proposed algorithm does not use approximation methods for the detected contour so that the outer corneal edge retains its individually variable shape. Therefore, special emphasis was put in the presented method on the most precise binarisation of the corneal profile. The method was compared with the known and used edge detectors: Sobel, Roberts and Canny operators.

## Results

The first step in assessing the correctness of the authors’ method for detecting the outer corneal edge in images from the Corvis^®^ ST tonometer was to check whether the detected contours contained any discontinuity points. Next, the contours detected by the new algorithm and those determined using the tested Sobel, Roberts and Canny operators were compared with outer corneal contours marked by the expert.

**Fig. 3:**
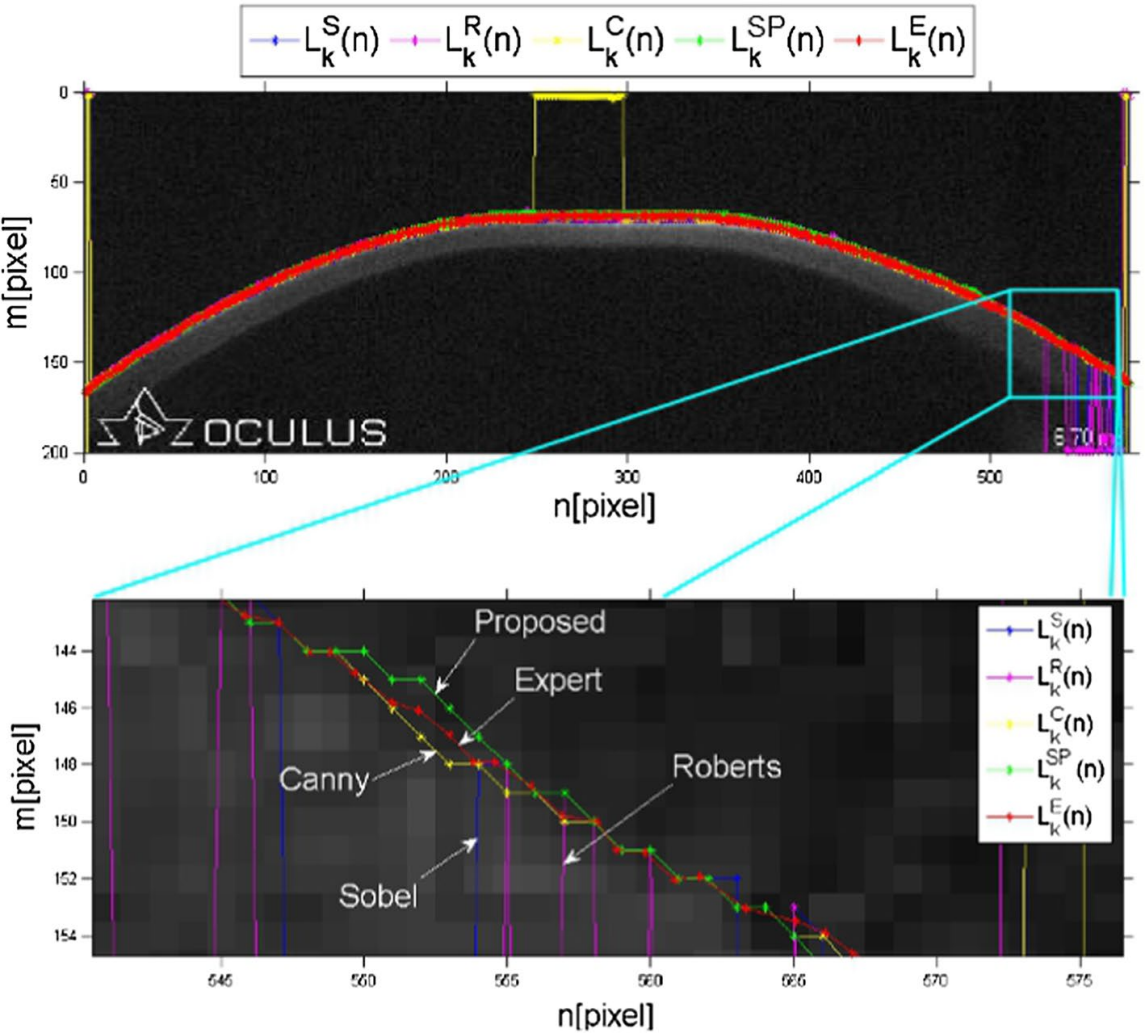
An image from the Corvis^®^ ST tonometer showing the outer corneal edge detected by means of the tested methods (Sobel—blue line, Roberts—magenta line, Canny—yellow line, proposed method—green line) together with the edge marked by the expert (red line)

The correctly determined outer corneal contour $L_{k}^{\text{SP}} \left( n \right)$ was an edge that did not contain any discontinuity points, and the difference in position between the contour points for subsequent images in the series was not greater than 5% of the number of image rows. In the case of the method proposed by the authors, the contour was detected correctly for approx. 90% of the 15,400 analysed 2D images. Figure 3 shows an image from the Corvis^®^ ST tonometer for the moment of the first applanation together with the contours of the outer corneal edge detected by means of the tested methods, i.e. the Sobel $\left( {L_{k}^{\text{S}} \left( n \right)} \right)$, Roberts $\left( {L_{k}^{\text{R}} \left( n \right)} \right)$, Canny $\left( {L_{k}^{\text{C}} \left( n \right)} \right)$ methods as well as the proposed new method $\left( {L_{k}^{\text{SP}} \left( n \right)} \right)$ and the external corneal contour marked by the expert $\left( {L_{k}^{\text{E}} \left( n \right)} \right)$.

The accuracy of the described outer corneal edge detection algorithms was determined based on the measurement error value $\delta_{k}$ (Eq. 1), calculated on the basis of the difference, obtained by a given method, in the position of the corneal edge and the position of the outer corneal contour designated by the expert. The expert’s work was computer-assisted, so it was possible to mark the edges for all 15,400 images.

1 $$\delta_{k} = \frac{1}{N} \cdot \mathop \sum \limits_{n = 1}^{N} \frac{{\left| {L_{k} \left( n \right) - L_{k}^{\text{E}} \left( n \right)} \right|}}{M}100\% ,$$

where $L_{k} \left( n \right)$—corneal contour determined by one of the analysed methods: Sobel operator, where: $L_{k} \left( n \right) = L_{k}^{\text{S}} \left( n \right)$, Roberts operator, where: $L_{k} \left( n \right) = L_{k}^{\text{R}} \left( n \right)$, Canny operator, where: $L_{k} \left( n \right) = L_{k}^{\text{C}} \left( n \right)$ or the authors’ method, where: $L_{k} \left( n \right) = L_{k}^{\text{SP}} \left( n \right)$, $L_{k}^{\text{E}} \left( n \right)$—corneal contour determined by the expert, *M*—number of image rows, *N*—number of image columns.

The measurement error was calculated for each of the four analysed methods separately and for all 15,400 images. The mean error values together with their minimum and maximum values as well as standard deviations obtained for each method are presented in Table 1.

**Table 1:** A summary of the mean error values $\delta_{k}$ and the minimum, maximum values and standard deviations of the error $\delta_{k}$ obtained for all the compared methods: Sobel, Roberts, Canny and the author’s (proposed) method of outer corneal edge detection in images from the Corvis^®^ ST tonometer

Method	*δ*_*k*_ (%)	*δ*_*k*(min)_ (%)	*δ*_*k*(max)_ (%)	std (%)
Proposed	0.16	0.09	3.62	0.19
Sobel	3.43	0.25	42.12	6.21
Roberts	5.78	0.17	61.67	9.19
Canny	1.26	0.53	50.70	3.11

The mean value of the measurement error ($\delta_{k}$) for the proposed method was the smallest and amounted to $0.16 \pm 0.19{\text{\% }}$. For individual patients (Table 2), this error did not exceed 1.25% and its minimum value was 0.11%.

**Table 2:** A summary of the minimum, maximum and standard deviation values of the error $\delta_{k}$ obtained for 110 patients for all the compared methods: Sobel, Roberts, Canny and the author’s (proposed) method of outer corneal edge detection in images from the Corvis^®^ ST tonometer

Method	*δ*_*k*(min)_ (%)	*δ*_*k*(max)_ (%)	std (%)
Proposed	0.11	1.25	0.13
Sobel	0.35	30.02	5.76
Roberts	0.26	47.45	8.66
Canny	0.61	28.36	2.78

**Fig. 4:**
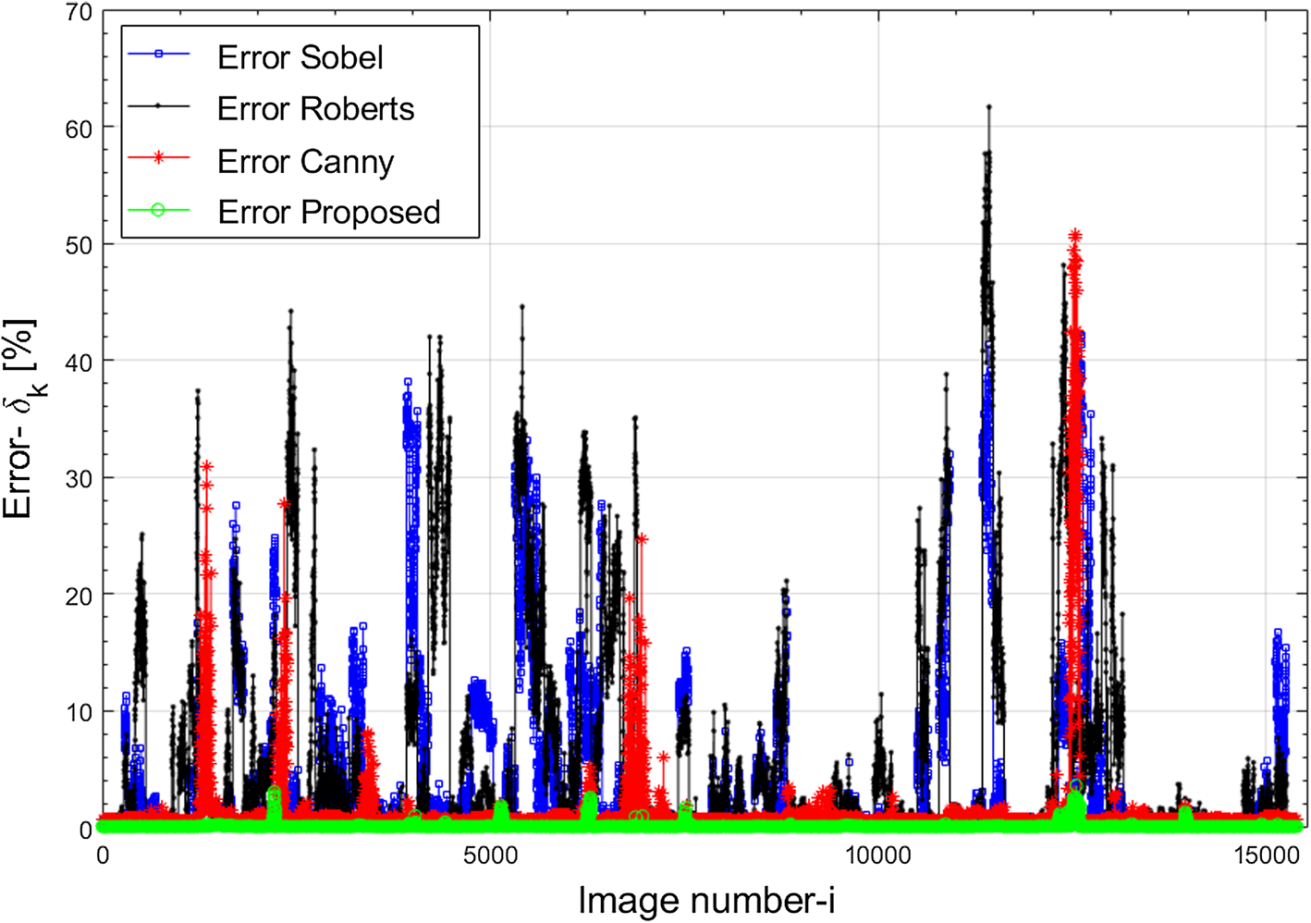
Graphs of values of the error $\delta_{k}$ for the individual methods: Sobel (blue), Roberts (black), Canny (red) and the authors’ method (green) of outer corneal edge detection, for each of the 15,400 analysed images

Taking into account the total of 15,400 images analysed, this error was not greater than 3.62%. The proposed method also provided the smallest minimum and maximum values of the error $\delta_{k}$ (the minimum value of $\delta_{k} = 0.09{\text{\% }}$ and the maximum—$\delta_{k} = 3.62{\text{\% }}$). On the other hand, the highest mean error value and standard deviation were recorded for the Roberts method: $5.78 \pm 9.19{\text{\% }}$. What is more, the largest error for all the analysed data of 61.67% and for individual patients equal to 47.45% was also in the case of Roberts edge detection. The highest error values result from the method used, which shows less resistance to local noise than the other edge operators. The most popular method is the Canny method, which has been modified many times for various applications in medical imaging. The disadvantage of the aforementioned method, in the analysed problem of detecting only the outer corneal border, is the detection of too many edges, including the edges of emerging artefacts—mainly light flares resulting from poor lighting. For the analysis of images from the Corvis^®^ ST tonometer, the mean value of the error $\delta_{k}$ for the Canny method is only 1.1% higher than the error for the proposed method. However, a large maximum error of 50.70% excludes the use of this method (in the presented version) in practice. Graphs of values of the error $\delta_{k}$ for individual methods, i.e. Sobel, Roberts, Canny and the proposed method of outer corneal edge detection, for each of the 15,400 analysed images are shown in Fig. 4.

**Fig. 5:**
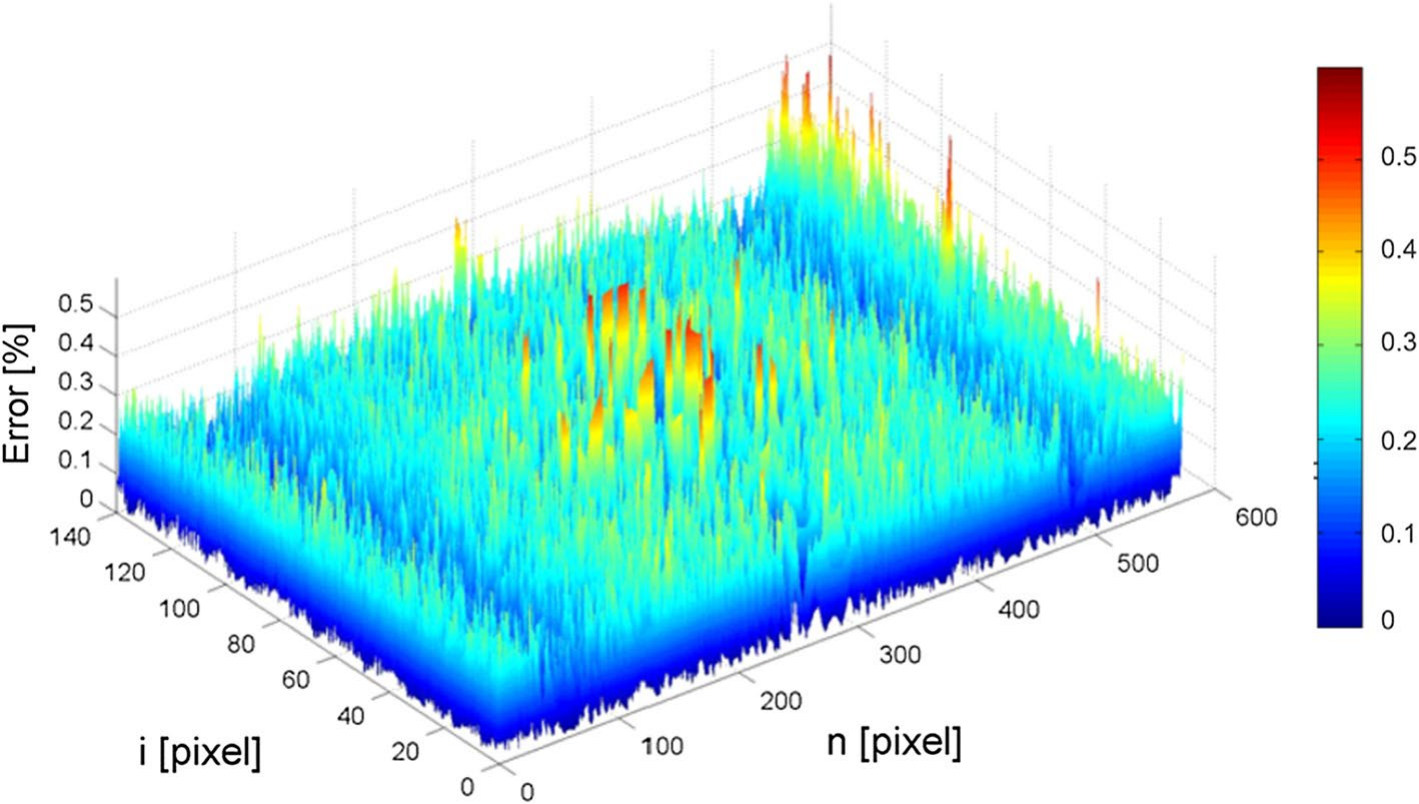
A three-dimensional graph of the mean error values of edge detection using the proposed method for one patient. The graph is presented in a jet colour map (ranges from blue to red, and passes through cyan, yellow, and orange)

A three-dimensional error graph for the proposed method for one of the patients is shown in Fig. 5. It presents the distribution of errors in the analysed images (repeated for all patients)—larger errors usually appear at the ends of the detected cornea and in its central area. The best results, i.e. the smallest distance between the curve detected by the authors’ method and the edge marked by the expert, can be observed in approx. $\frac{1}{6}$ and $\frac{5}{6}$ length of the cornea (mm). Such distribution of errors results from the dynamic corneal deformation, when the cornea changes its shape the least at the mentioned points.

In the literature, other methods for detecting the outer corneal edge in images from the Scheimpflug camera are also suggested. Due to the various purposes of the studies quoted, the methods of testing and validating the suitability of the algorithms proposed by other authors [13, 32, 43] are also different (Table 3).

**Table 3:** A comparison of the tested methods of outer corneal edge detection in images from the Corvis^®^ ST tonometer, i.e. Sobel, Roberts, Canny methods and the authors’ (proposed) method of outer corneal edge detection, with the methods proposed by other authors

Outer edge detection method	Verification parameter			Number of analysed patients
	$\delta_{k}$	*r*	$\Delta A\;{\text{time}}$	
Proposed	$0.16\%$	−	−	110 healthy subjects (110 eyes)
Sobel	$3.43\%$	−	−	
Roberts	$5.76\%$	−	−	
Canny	$1.26\%$	−	−	
Ji et al. [13]	−	0.9943	−	40 eyes (normal group)
Ji et al. [13]	−	0.9972	−	30 eyes (keratoconus group)
Kasprzak et al. [43]	−	−	$\pm \;23$ ms	10 healthy subjects (only one eye was examined for each patient, 9 measurements were made for each eye)

$\delta_{k} \left[ \% \right]$—Measurement error (Eq. 1), calculated based on the difference between the position of the corneal edge obtained by a given method and the position of the outer corneal contour determined by the expert

*r*—Pearson’s correlation coefficient. The parameter values (*r*) are calculated for the correlation analysis of the method proposed by Ji et al. [13] with the manual method (expert). The correlation was determined for the Peak Distance parameter describing the distance between two corneal peaks at the moment of the largest corneal concavity

$\Delta A\;{\text{time}}$ (ms)—The difference between corneal applanation times determined using the built-in method (available in the Corvis^®^ ST tonometer software) and the method proposed by Kasprzak et al. [43]

It is also worth noting that in the practical analysis of the errors presented, it is important to determine the purpose of the Corvis^®^ ST tonometry tests. If it is to show the total dynamic corneal deformation and its speed, analyse its vibrations etc., the outer corneal border in each of the 140 images (constituting the full examination of 1 patient) must be detected correctly. Especially, in such cases, the value of the presented error is significant. It is worth noting that despite obtaining small errors of outer edge detection (for individual patients $\delta_{k} = 0.11 \pm 0.13{\text{\% }}$), the possibility of enhancing outer contour detection (obtaining smaller values of the error $\delta_{k}$) would improve the accuracy of ophthalmologic diagnosis. This is due to the fact that the cornea (especially the diseased one) subjected to dynamic changes behaves in an unconventional way. That is why there is no standardised biomechanical model of the cornea (for dynamic conditions). Therefore, the more precise the detection of the outer corneal edge, the more accurate and reliable the assessment of corneal biomechanics, and thus the ophthalmologic diagnosis. A different situation is the analysis of corneal phenomena such as: the length of the first and second applanation, the measurement of the maximum corneal deformation (highest concavity HC), when the upper corneal edge must be correct for a specific image corresponding to a given event.

## Discussion

The paper presents a fully automatic method for outer corneal edge detection in images from the Corvis^®^ ST tonometer. The method was compared with commonly known edge detectors: Roberts, Sobel and Canny operators. The selected operators are classic edge detection methods, the most popular and widespread in the literature. The proposed methodology is characterised by repeatability and accuracy ($\delta_{k} = 0.16 \pm 0.19{\text{\% }}$). Outer corneal edge detection is an indispensable step to acquire numerical parameters, calculated on the basis of data from the Corvis^®^ ST tonometer, relevant in terms of their practical use in ophthalmology. Therefore, the accuracy of the algorithm responsible for this process is an extremely important issue. It should be noted that the proposed method is not the only possible approach to solving the analysed problem. For this purpose, it is also possible to apply other methods used in medical image processing. However, each of the new methods must be adapted to individual processing needs—in this case, corneal edge detection.

The problem of outer corneal contour detection was mentioned in the paper [32], where the authors Koprowski et al. presented two approaches to corneal edge detection. The first proposed method was based on Otsu segmentation. The correctness of the method was about 80% for 13,400 analysed images. The next presented algorithm was based on the well-known Canny edge detection method and morphological image transformations. Here, Koprowski et al. achieved 90% correctness for the same image database.

A completely different approach to the problem of determining the corneal edge was presented by Ji et al. [13]. The edge detection methodology was based on a three-step algorithm: removing artefacts, creating phase-based images and marking the corneal edge. The correctness of this method was compared with the results of segmentation of the built-in method, provided with the Corvis^®^ ST device, as well as manual segmentation (expert). In the analysed research group, 40 healthy eyes and 30 eyes with keratoconus were tested, which together gave 9800 2D images. The reference parameters for all the algorithms analysed in paper [13] were the values of the central corneal thickness (CCT) and the distance between the two peak points at the time of maximum corneal concavity (peak distance, PD). Correlation analysis (Bland–Altman test and Pearson’s correlation coefficient) showed a strong correlation between the presented method and manual segmentation ($p \le 0.01$, two-sided *T* test). However, there was no strong correlation with the built-in method (during the corneal deformation stage: $p = 0.389$ for healthy patients, $p = 0.222$ for patients with keratoconus). Nevertheless, the method of Ji et al. turned out to be more robust in the case of images with noise and artefacts (characteristic and numerous in images from the Corvis^®^ ST tonometer) compared to the built-in method, which could not handle such cases.

The determination of corneal contours was also an indispensable stage in the study by Rogowska et al. [44], where the influence of age on changes in corneal deformation was investigated. For the segmentation of both the outer and inner edges, the Otsu thresholding method was used, followed by approximation of the contours using the sixth grade Chebyshev polynomial. Due to the increase in noise and small stabilisation of the polynomial fit on the edges of the designated corneal profiles, ultimately 10% of the data was cut off from each side of the image. As in the above-mentioned studies [13, 32], edge detection was only a step leading to the proper analysis being the subject of this paper. However, it is worth noting, that it was a necessary stage without which it would be impossible to examine the dynamic corneal parameters. Rogowska et al. also pointed out that 10% data trimming resulted in different results than in the case of analysis for profiles with a 5% cut-off. The above may indicate that the analysis of the incomplete corneal contour may introduce an error in the conducted tests, and the spherical shape of the cornea also affects the obtained results.

As shown in the above comparisons, the method presented in this paper indicates the highest accuracy of edge detection; the measurement error for the 15,400 analysed 2D images was: $\delta_{k} = 0.16 \pm 0.19{\text{\% }}$. It should be emphasised that the results obtained are influenced by the adopted exclusion criteria, which eliminated all unusual cases. In addition, it is worth considering other factors affecting the repeatability and reproducibility of the results obtained, among others, test conditions (temperature, lighting), changes in the position of the patient’s head, various technological parameters of Corvis^®^ ST tonometers.

Owing to the proposed method, it was possible to obtain small error values, and thus increase the accuracy of measurements, which will allow for precise diagnosis. Obtaining the smallest possible measurement error is particularly important in this case because the corneal deformation process is dynamic, covering not only the cornea itself but also the entire eyeball. Therefore, even relatively small changes in acquired parameters (whose determination requires the localization of the outer edge) may affect the accuracy of diagnosis. The proposed method can be used as the first procedure in determining the dynamic parameters of the cornea available in the Corvis ST tonometer software. Thus, it can improve the commercially available tool.

In subsequent research, on the basis of acquired corneal contours, the authors want to analyse the dynamic corneal deformation process and related phenomena. Ultimately, owing to the information obtained, it is planned to perform automatic classification (verification) of patients who have been diagnosed with keratoconus. The possibility of testing the proposed solution for data from different devices and for patients coming from outside Europe would also be interesting and valuable. It would enable to check the repeatability and stability of the method.

The applied image processing techniques used as well as other currently used methods [46–48] invariably require individual profiling and adjustment of acquired parameters depending on the analysed biomedical data set.

## Conclusions

The use of an ultra-fast Scheimpflug camera in the modern Corvis^®^ ST tonometer has opened new possibilities in the field of analysis of biomechanical parameters of the eye and the dynamic deformation process occurring during tonometry tests. In this study, the authors addressed the basic problem being one of the first, and at the same time, key stage in the analysis of corneal images—the detection of the outer corneal edge in the full sequence of 140 images from the Corvis^®^ ST tonometer.

Over 15,400 two-dimensional images acquired from 110 patients using the Corvis^®^ ST tonometer were analysed. A new, fully automatic method for detecting the outer corneal contour was proposed. Its operation was compared with three edge detectors, commonly used in the analysis of medical images, i.e. Roberts, Sobel and Canny operators. The authors obtained an error of the proposed method at the level of 0.16% and its high repeatability (standard deviation 0.19%). For the other methods, the errors were $5.78 \pm 9.19{\text{\% }}$, $3.43 \pm 6.21{\text{\% }}$, and $1.26 \pm 3.11{\text{\% }}$ for the Roberts, Sobel and Canny operators, respectively. The above indicates, among others, that the presented algorithm is more resistant to noise characteristic of Corvis^®^ ST tonometry tests. The analysis was carried out in MATLAB^®^ 9.0.0.341360 (R2016a).

The proposed method for detecting the outer corneal edge can be used in the analysis of dynamic parameters of the cornea. It increases the accuracy of measurements and, thus, can be the basis for creating another precise diagnostic tool for patients with eye surface diseases.

## Methods

### Materials

The images used for the analysis come from the Corvis^®^ ST tonometer. They were exported directly from the software provided with the device (version 1.0r38 rev. 821) as a sequence of 140 *.jpg format images (available formats are: *.U12, *.cst, *.avi, *jpg). The images have the resolution $M \times N = 200 \times 576$ pixels (where *M*—number of image rows, *N*—number of image columns). The images are from patients undergoing routine eye examinations aimed at controlling intraocular pressure. The study group included 110 patients, 63 women and 47 men, aged 21–81 years. The mean age of subjects was 61 years and they were all European. Previous surgical treatments, retinal detachment, and peripheral hypertension were factors excluding patients from the study group. The other exclusion criteria were as follows: diseases and changes of the cornea that could affect the thickness and flexibility of the tissue, such as corneal softening, ulceration, threatening or performed corneal perforation, scars, conditions after injury of the ocular surface and after ophthalmic surgical treatment, systemic diseases with ocular manifestation (diseases of the connective tissue, skin and mucous membranes), refractive errors (± 4.0 spherical dioptres and ± 2.0 cylindrical dioptres) and long-term topical use of drugs that can damage the cornea (mainly medicines with preservatives). All 110 eyes (left or right eyes) were examined and 15,400 2D images were obtained for analysis. The research was carried out in cooperation with doctors by Sven Reisdorf, a specialist from the Oculus laboratory (OCULUS Optikgeräte GmbH, Wetzlar, Germany). The tests were performed in accordance with the Declaration of Helsinki, the data were anonymised and healthy patients gave their voluntary consent.

### Methods of outer corneal edge detection

The well-known edge detectors commonly used in medical images analysis were used: Roberts, Sobel and Canny operators. The above operators were selected for comparative analysis because they are one of the most popular methods among those cited in the literature. Moreover, the tested algorithms (Canny, Sobel, Roberts), due to their versatility and simplicity of operation, are repeatedly used as methods of fast and effective edge detection in images. In addition, the proposed method of edge detection is described.

### Image preparation for analysis (pre-processing)

Images were obtained directly from the Corvis^®^ ST tonometer as a sequence of 140 2D images $L\left( {m,n} \right),$ where *m*—number of rows $m \in \left( {1, 200} \right)$, *n*—number of columns $n \in \left( {1,576} \right)$. The algorithm was written in MATLAB^®^ version 9.0.0.341360 (R2016a) using the Image Processing Toolbox (version 9.4) and the Neural Network Toolbox (version 9.0).

The first stage of analysis was median filtration. The size of the filter mask was selected based on the measurements and the mean value of the measurement noise. The observed noise was mainly caused by the noise of the CCD converter in the form of white pixels with brightness exceeding 80%. Considering the above, it was found that the largest noise had an area of no more than 24 pixels. Thus, a $7 \times 7$ pixel mask *h* was adopted. Then, the whole image was normalised so that the brightness values in the resulting image $L_{\text{F}} \left( {m,n} \right)$ ranged from 0 to 1.

### Image processing

The filtered and normalised image $L_{\text{F}} \left( {m,n} \right)$ formed the basis for main transformations aimed at determining the outer corneal edge.

### Known edge detection methods

Edge detection was carried out successively using the three most popular operators for edge detection: Roberts, Sobel [49, 50] and Canny [51]. The edge detection methods selected for analysis belong to the category of gradient operators. Gradient methods detect edges by searching for the maximum and minimum in the first derivative of the image.

The Sobel method [52] locates edges using the Sobel approximation to the derivative. It precedes the edges at the points with the highest gradient. In the Sobel technique, a 2D spatial gradient quantity is performed in an image and, as a result, regions of high spatial frequency that correspond to edges are highlighted. It is generally used to find the estimated absolute gradient magnitude at each point in an input grayscale image. The operator is conjectured to consist of a pair of 3 × 3 complication kernels. One kernel is simply the other one rotated by 90°.

The Roberts method [52] performs simple, quick to compute, 2D spatial gradient measurements in an image. The method highlights regions of high spatial frequency that often correspond to edges. Pixel values at every point in the output represent the estimated complete magnitude of the spatial gradient of the input image at that point. It is very similar to the Sobel operator.

The Canny edge detection algorithm [53] is known as the optimal edge detector. The algorithmic steps are as follows:

- Image convolution with a Gaussian function to obtain a smooth image.
- Application of the first difference gradient operator to compute edge strength, then edge magnitude and direction.
- Application of non-maximal or critical suppression to the gradient magnitude.
- Application of the threshold to the non-maximal suppression image.

For the Roberts method, a threshold of 0.03 was used; for the Canny method, the applied threshold value was 0.1, and the standard deviation of the Gauss filter was set at 0.99. In the case of the Sobel method, the best results were obtained for the automatic threshold, determined in a heuristic manner based on the estimated RMS value of the measurement noise. Automatic selection of threshold values for this method is described in detail in paper [54]. The images resulting from the edge detection operation are as follows: $L_{\text{R}} \left( {m,n} \right)$, $L_{\text{S}} \left( {m,n} \right)$, $L_{\text{C}} \left( {m,n} \right)$.

**Fig. 6:**
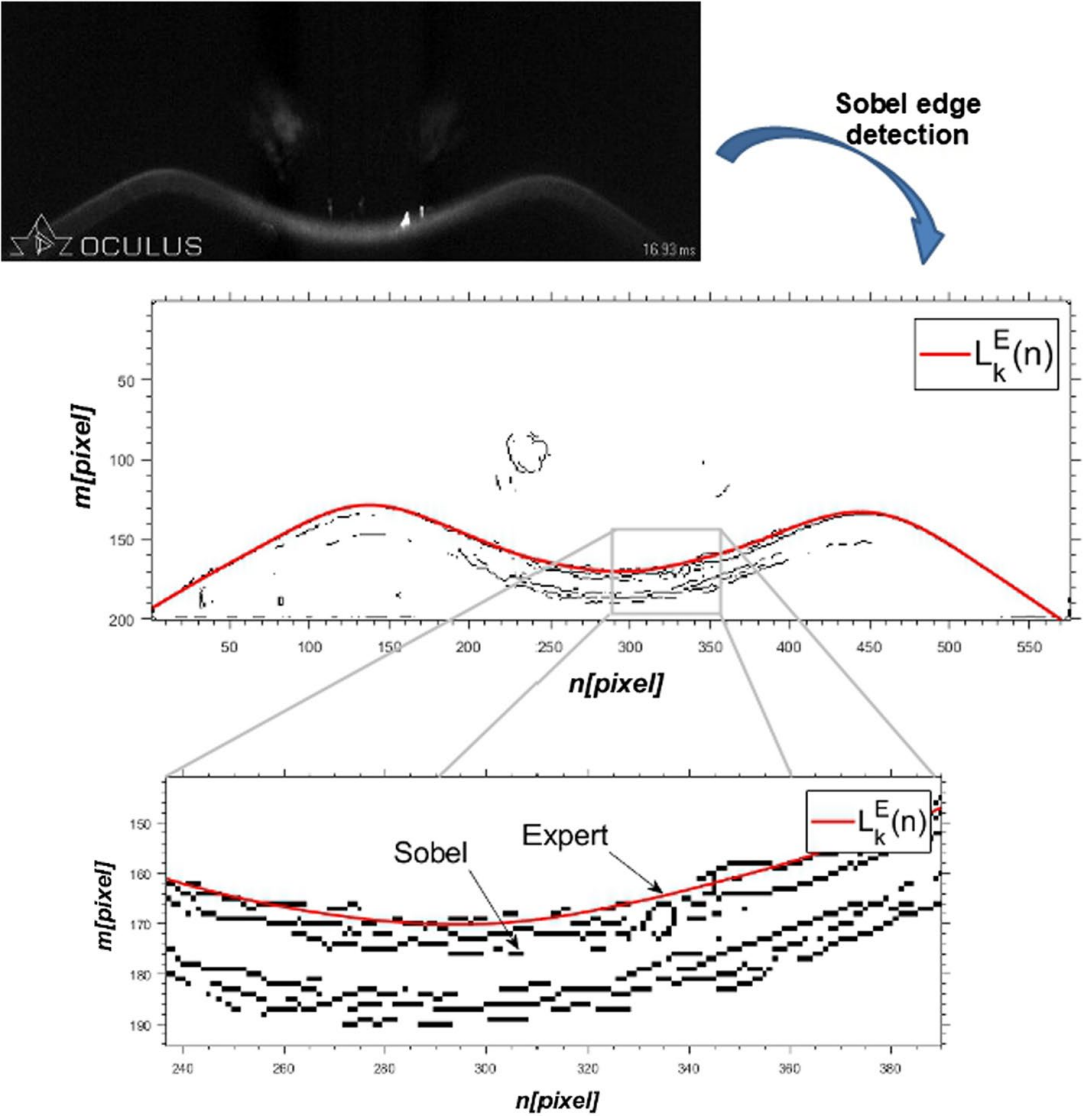
The result of Sobel edge detection together with the outer corneal edge marked by the expert $\left( {L_{k}^{\text{E}} \left( n \right)} \right)$

Figure 6 shows the selected image $L_{\text{S}} \left( {m,n} \right)$ for the characteristic moment of the deformation process: maximum corneal deformation (HC) along with the corneal edge marked by the expert (the expert’s work was computer-assisted).

In addition, an analysis was also carried out for the method presented in the paper by Koprowski et al. [32], based on edge detection by Canny operator, as well as for the active contour method [45]. Apart from applying the classic Canny operator (as in this paper), the solution presented in [32] also uses morphological image operations, which provided 80% efficiency.

Another tested method (active contour method) [45] implements segmentation by gradually transforming a closed contour in such a way that it finally adheres closely to the borders of the selected object. The contour is transformed based on the function values of the contour energy, which consists of regulatory constraints, associated with the need to maintain contour continuity and find the shortest contour, image appearance parameters and additional restrictions. Among the image parameters, both local features, e.g. related to the image gradient, and global features, e.g. texture, calculated inside and outside the contour are used. An important issue of this method is the initialization of the contour. For both solutions based on the image gradient and methods using global features, prior knowledge about the location of objects in the analysed image is required. The use of this approach has proved problematic in the case of outer corneal contour detection in images from the Corvis^®^ ST tonometer, because this method requires the assumption of a fixed position of the cornea in the image, which is impossible for the examined images. This is due to the specifics of tonometric examinations, in which the cornea deforms dynamically. Furthermore, during the examination the patient can move, which additionally changes the position of the cornea in the analysed images. Given the above, this method was not used in further research.

Edge detection methods based on Otsu segmentation are also popular in the literature [55]. This method assumes that the histogram of the segmented image consists of two normal distributions, each of which represents one of the pixel classes in the image, i.e. the object pixel class and the background pixel class. The purpose of segmentation in the Otsu method is to allocate image pixels to one of these classes; so, this is an example of segmentation by binarisation. This task is accomplished by finding an optimal threshold that maximises the segmentation quality assessment indicator in the form of inter-class variance. This method, however, was not used by the authors in the present study due to the expected unsatisfactory results supported by the results obtained in another study [32].

For the applied edge operators and other tested methods proposed in papers [13, 43, 56, 57], the following problems appear in the analysed problem:

- Each of the applied edge detection operators marked more than one edge.
- Without the expert’s assessment, it is impossible to select the edge that corresponds to the outer corneal contour.
- There are numerous discontinuities in the detected edges.
- Edges of objects not belonging to the outer corneal contour are detected, e.g. iris, light flares that are artefacts resulting from bad lighting, and others.

Due to the fact that, as shown in Fig. 6, edge detectors provide images containing not only the outer corneal edge, only the first pixels with the value ‘1’ (for each column) were taken for further analysis, thus obtaining—for the Sobel operator, the edge: $L_{k}^{\text{S}} \left( n \right)$, for the Roberts operator—$L_{k}^{\text{R}} \left( n \right)$, and for the Canny operator—$L_{k}^{\text{C}} \left( n \right)$. This criterion is based on the assumption that the outer corneal edge should be the first edge detected by the applied operators.

### Proposed algorithm

**Fig. 7:**
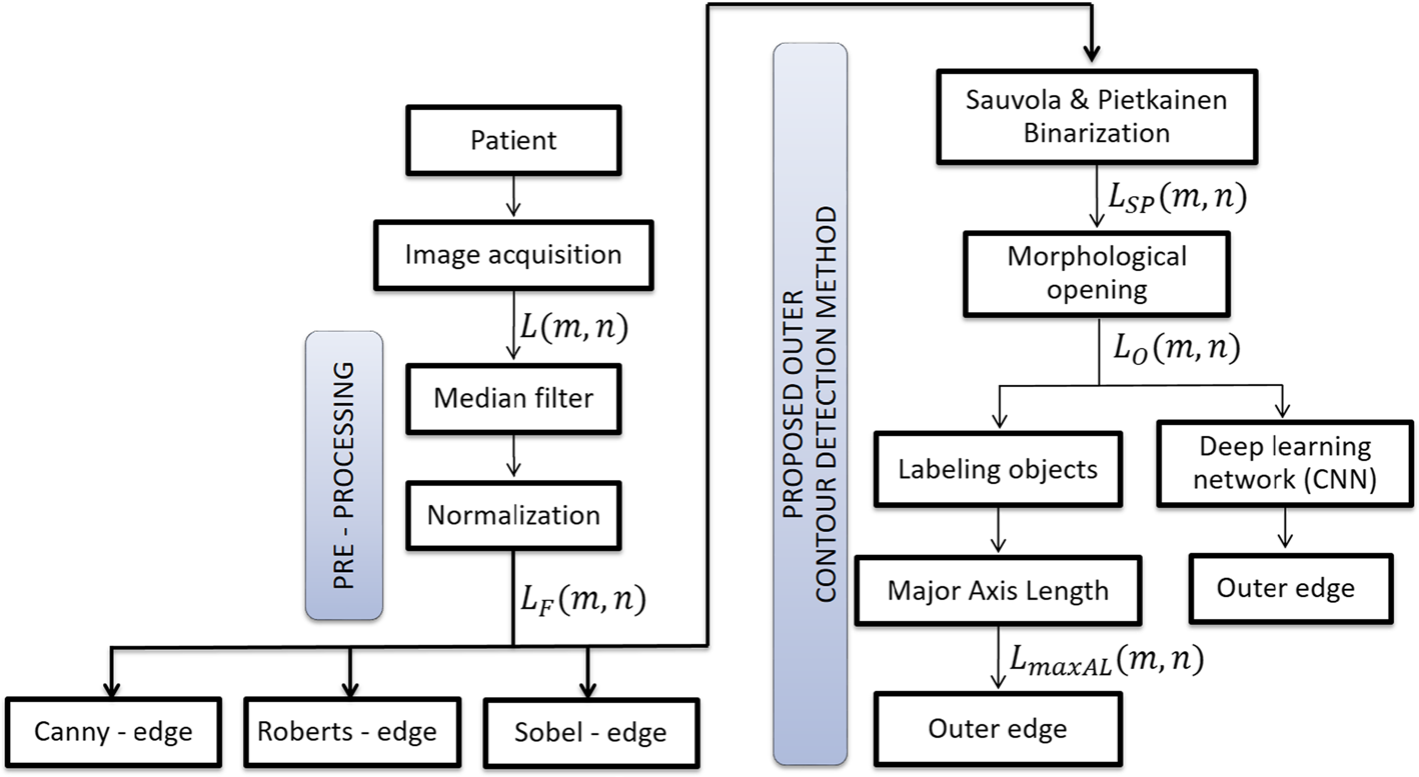
A block diagram showing individual stages of analysis. During image pre-processing, data were prepared to detect the outer corneal edge. In the subsequent stages of data processing, the known edge detection methods were used and the proposed new method of outer corneal contour detection in images from the Corvis^®^ ST tonometer was presented

The proposed new method for detecting the outer corneal edge is based on local thresholding using Sauvola and Pietkainen’s method [58] and the authors’ algorithm. The individual stages of data processing for the proposed new method as well as for known methods are shown in the block diagram (Fig. 7).

According to the methodology described in paper [58], the value of the binarisation threshold $t\left( {m,n} \right)$ was determined on the basis of the mean $\mu \left( {m,n} \right)$ and standard deviation $\sigma \left( {m,n} \right)$ of the pixels in a window sized $w \times w$:

2 $$t\left( {m,n} \right) = \mu \left( {m,n} \right)\left[ {1 - k\left( {\frac{{\sigma \left( {m,n} \right)}}{R} - 1} \right)} \right],$$

where $t\left( {m,n} \right)$—the value of the set threshold, for the pixel with coordinates $\left( {m, n} \right)$, $m \in \left( {1,200} \right)$, $n \in \left( {1,576} \right)$, $\mu \left( {m,n} \right)$—mean brightness for a given window, $\sigma \left( {m,n} \right)$—standard deviation for a given window, $k$—constant $k > 0$, selected in an experimental way $(k = 0.25$), $R$—maximum standard deviation.

The window size $w \times w$ was chosen directly for the data and is 75 × 75 pixels for each of the analysed images. The *k* parameter enables to control the binarisation threshold value in the local window. There is no consistency in the literature as to the best value for this parameter. In the study by Sauvola et al. [58], $k = 0.5$ was used; in the study by Rangoni et al. [59], the *k* parameter was 0.4; whereas, Badekas et al. [60] chose $k = 0.34$ as the most optimal value. The selection of the *k* parameter value is, therefore, strictly dependent on the data. In addition, as already pointed out by other authors [58], the algorithm itself is not very sensitive to changes in the values of *k*. For the case presented in this paper, the conducted experiments showed that the best results were obtained for $k = 0.25$.

To optimize the calculation time of the mean values of brightness and standard deviation in a given window, integral images were used [61, 62]. For the original image $L\left( {m,n} \right)$, an image $L_{i} \left( {m,n} \right),$ being the integral image representation of $L\left( {m,n} \right),$ was introduced. The value of the integral image at any point $\left( {m,n} \right)$ of the image is the sum of the pixel values above and to the left of the pixel with the coordinates $\left( {m, n} \right)$ of the original image $L\left( {m,n} \right)$:

3 $$L_{i} \left( {m,n} \right) = L_{i} \left( {m - 1,n} \right) + L_{i} \left( {m,n - 1} \right) - L_{i} \left( {m - 1, n - 1} \right) + L\left( {m,n} \right)$$

The values of coordinates outside the image frame are 0:

$$L_{i} \left( {m - 1,n} \right) = 0\;{\text{when }}m = 1$$

$$L_{i} \left( {m,n - 1} \right) = 0\;{\text{when}}\;n = 1$$

$$L_{i} \left( {m - 1, n - 1} \right) = 0 \;{\text{when}}\;m = 1, n = 1$$

Using an integral image, it is possible to determine, in an efficient and quick way, the sum of pixel brightness in a given window sized $w \times w$, and then the desired local mean $\mu_{i} \left( {m,n} \right)$.

**Fig. 8:**
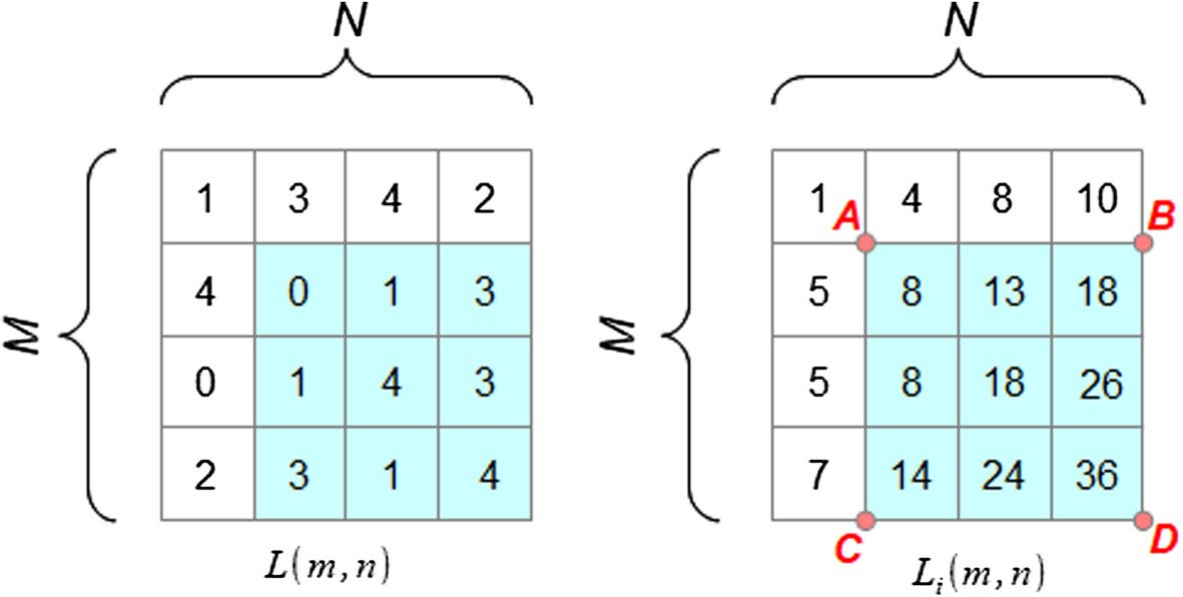
The sum of pixels in the shaded square ABCD, with the coordinates $A\left( {m - w,n - w} \right)$, $B\left( {m - w,n} \right)$, $C(m,n - w)$, $D\left( {m,n} \right)$, representing the window sized $w \times w$, can be calculated using the value of the integral image $L_{i} \left( {m,n} \right)$ as follows: $D + A - B - C = 36 + 1 - 10 - 7 = 20$

For the example shown in Fig. 8, the mean in a window sized $3 \times 3$ (in the figure the window is marked in blue) can be determined based on the following formula:

4 $$\mu_{i} \left( {m,n} \right) = \frac{1}{{w^{2} }} \cdot \left( {L_{i} \left( D \right) + L_{i} \left( A \right) - L_{i} \left( B \right) - L_{i} \left( C \right)} \right)$$

The integral image can be used in a similar way to determine standard deviations.

Sauvola and Pietikainen’s binarisation with the local threshold $t (m,n)$ provided the image $L_{\text{SP}} (m,n)$, which required further correction. For this purpose, a morphological opening operation with a disc-shaped structural element SE with a 3-pixel radius was used. For monochrome images and symmetric structural elements, this operation can be written as:

5 $$L_{o} (m,n) = \mathop {\max }\limits_{\text{SE}} \left(\mathop {\min }\limits_{\text{SE}} \left(L_{\text{SP}} (m,n)\right)\right)$$

To extract only the corneal image, all the objects present in the binary image $L_{\text{O}} \left( {m,n} \right)$ were labelled.

**Fig. 9:**
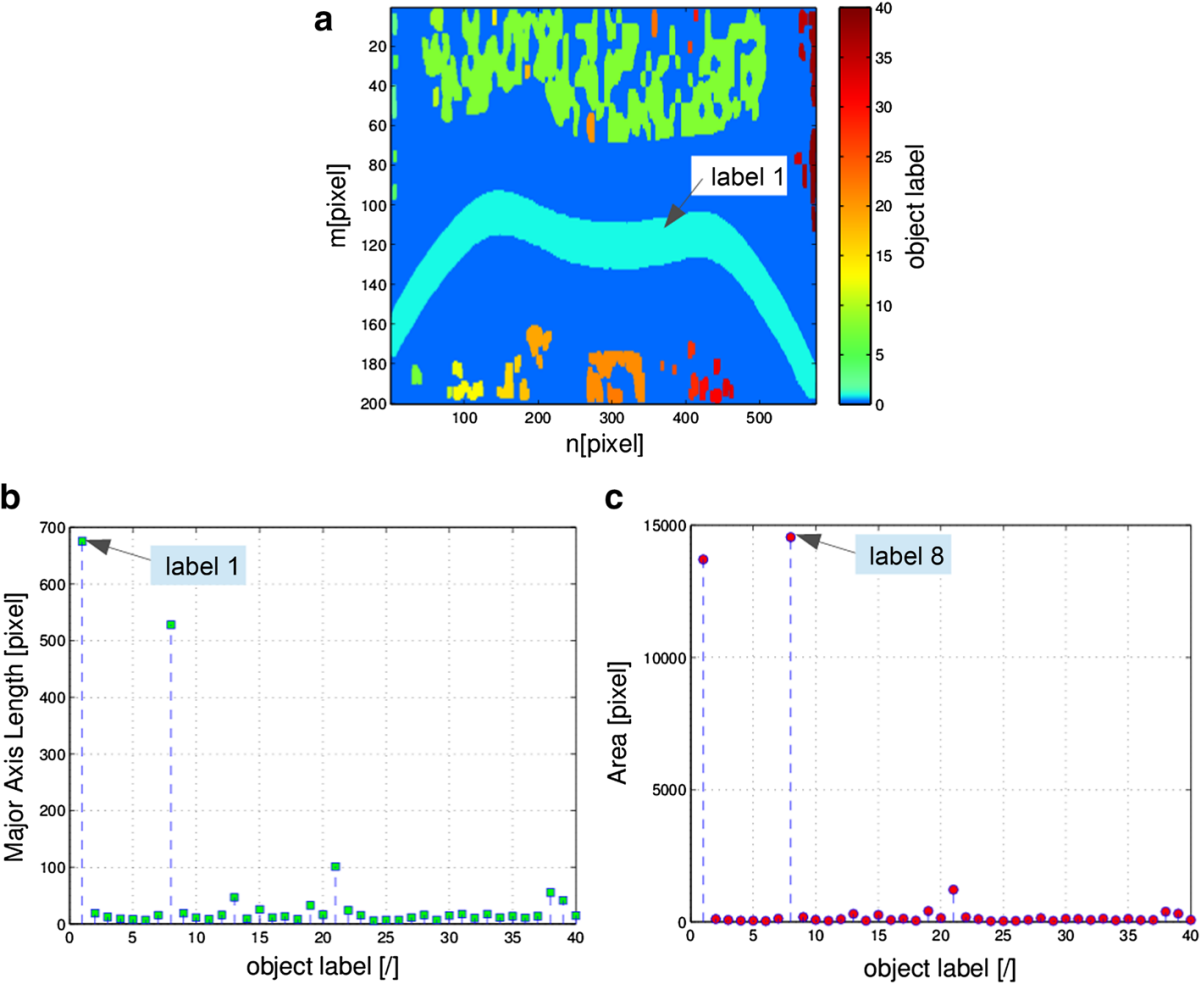
A comparison of characteristic features of the labelled objects in a selected corneal deformation image. **a** The image $L_{\text{O}} \left( {m,n} \right)$ after labelling objects. Elements described by particular labels were assigned colours from the artificial colour map. The index of 1 corresponds to the area of the cornea. **b** A graph showing major axis lengths of the labelled objects. The largest parameter value belongs to the object with the label 1 (this area corresponds to the cornea). **c** A graph showing surface areas of the labelled objects. The highest parameter value belongs to the object with the label 8 (this area does not represent the cornea)

Then, on the basis of a comparative analysis of the characteristic features of the labelled objects, one feature was selected, i.e. major axis length, allowing for automatic and unambiguous classification of the object being a binary image of the cornea. For this purpose, the areas and major axis lengths of the previously determined objects were compared. Major axis length was defined as the length (in pixels) of the major axis of the ellipse that had the same normalised second central moments as the region. For all of the images analysed, the object with the largest major axis length (as opposed to the object with the largest area) always corresponded to the binary corneal image (Fig. 9).

Based on the image $L_{\text{maxAL}} \left( {m,n} \right)$ (representing the cornea) obtained after applying the above-described criterion, the outer corneal edge $L_{k}^{\text{SP}} \left( n \right)$ was determined, defining it in the same way as for the methods described in “Known edge detection methods” subsection, as the first pixels having the value ‘1’ for each column. The comparison of the outer corneal contour detection method using the major axis length parameter with known edge detection methods is presented in “Results” section.

Characteristics that allow for corneal image detection in a binary image can be searched for with the use of deep learning methods that provide much greater accuracy of the analysed task and efficiency in making decisions based on data analysis. For the case under study, a database of 150,000 2D images with a resolution of 200 × 576 pixels was created containing a binary corneal image of the entire deformation process and individual areas constituting noise, visible in the image $L_{\text{O}} \left( {m,n} \right)$. To recognise corneal images, a convolutional neural network (CNN) was used, which allowed for both the detection of features and the classification itself (recognition of the corneal image). For the given problem, 9000 images from each of the two categories were randomly selected, i.e. 9000 images of the cornea and 9000 images containing other non-corneal objects. Training and test sets were created (2/3 of the data formed a training set and 1/3—test set). All images were allocated to each set at random and were not duplicated. It is, therefore, possible that images from the same patient will be in both the test and training set. The corneal images for a given patient and among other patients are quite similar; therefore, no additional rigour was introduced to prevent the presence of images from one patient in both sets. On the other hand, images showing areas that are not corneas are very diverse and generally there are no disturbances characteristic only for a given patient, which would introduce the possibility of network overtraining.

The trained accuracy of the model was 100% (validation frequency = 100%). The defined network consisted of 15 layers, including three convolution layers with 3 × 3 filters. The number of filters in the convolution layers was 8, 16 and 32, respectively. The applied activation function was a rectified linear unit (ReLU). Pooling with a 2 × 2 rectangular region was also used. Classification with such a trained neural network provided 100% accuracy (for the test set). To the best of the authors’ knowledge, this is the first study presenting the possibility of using deep learning in the discussed issue. The use of CNN in the studied issue was aimed at presenting an alternative solution, whose advantages can be fully appreciated in studies involving a much larger research group. The comparison of both presented solutions for detecting an image containing only the cornea, i.e. detection using the major axis length parameter and the solution using CNN, will be presented in authors’ subsequent papers.

## Data Availability

Not applicable.

## Abbreviations

IOP: intraocular pressure
CCT: central corneal thickness
ORA: Ocular Response Analyzer
CH: corneal hysteresis
CRF: cornea resistance factor
HC: highest concavity
DA: deformation amplitude
OCT: optical coherence tomography
CNN: convolutional neural network
